# Water stress reduces cellulose deposition in the cell wall and increases wax content, resulting in decreased fiber quality

**DOI:** 10.3389/fpls.2025.1611390

**Published:** 2025-07-01

**Authors:** Yongchao Han, Yi Yang, Han Luo, Jinhui Cui, Bifu Kuang, Xinyu Zhang, Jie Sun, Jian Wei Xu, Feng Liu

**Affiliations:** Key Laboratory of Oasis Eco-agriculture, College of Agriculture, Shihezi University, Shihezi, Xinjiang, China

**Keywords:** water deficit, cellulose, sucrose, cuticular wax, fiber quality

## Abstract

**Introduction:**

Water deficiency reduces cotton fiber quality, but the underlying mechanisms behind this decline remain poorly understood. Although the cuticle is critical for plant water homeostasis under drought, few studies have addressed the relationship between water stress, fiber epidermal wax, and fiber quality. Thus, studying the interaction between fiber cuticular wax and quality is crucial for understanding plant drought tolerance and breeding superior drought-resistant cotton varieties.

**Methods:**

This experiment was designed as a randomized block design. Two cotton cultivars, Xincaimian7 (XC7, with high cuticular wax) and Shidamian217 (SD217, with low cuticular wax), were selected as materials. Two irrigation regimes were applied: well-watered (WW) and water-deficit (WD), each with three independent biological replicates.

**Results:**

Results showed WD irrigation significantly reduced the cotton fiber accumulation rate, particularly between 25-30 days post-anthesis (DPA). Compared with WW irrigation, the rate decreased by 23.62% and 30.82% respectively. WD treatment significantly inhibited the expression of the genes encoding sucrose synthase *GhSusy* and cellulose synthase *GhCesA* in cotton fibers. At 30 DPA, compared to the WW treatment, the sucrose contents in SD217 and XC7 fibers decreased by 18.66% and 12.85%, while cellulose contents dropped by 9.91% and 17.17%, respectively, resulting in a significant decrease in the thickness of the cell walls by 10.59% and 9.50% respectively. However, the WD treatment significantly induced the expression of wax synthesis-related genes in cotton fibers. Compared with the WW treatment, at 30 DPA, the epidermal wax contents of the fibers of SD217 and XC7 increased significantly by 81.87% and 97.34%, respectively. Correlation analysis reveals a significant positive relationship between fiber strength, length, and the contents of cellulose and sucrose (*p<0.01*). Conversely, a significant negative correlation exists between these fiber properties and wax content (*p<0.01*).

**Discussion:**

In summary, WD reduces the sucrose content in cotton fibers and induces wax accumulation. Thinner cell walls combined with a thicker wax layer altered the mechanical properties of the fibers, thus leading to a decrease fiber quality. Therefore, when breeding drought-tolerant varieties, breeders need to balance the drought resistance with the sucrose and wax characteristics of the fibers.

## 1 Introduction

Cotton is an important industrial crop. Due to the highly specialized characteristics of cotton fiber, it has unique economic value in the textile industry. However, cotton yield and fiber quality are negatively affected by abiotic stress factors (Chalise et al., 2022). Studies have shown that water stress has a significant negative impact on cotton yield (Basal et al., 2014; Yilmaz et al., 2021; Bista et al., 2024). Our recent research found that the long-term drought in the growing season reduces cotton yield by up to 50% (Han et al., 2025). Drought also has negative effects on fiber length, strength and maturity (Hu et al., 2018; Abdelraheem et al., 2019; Gao et al., 2021; Chalise et al., 2022). Cotton fibers are single-celled structures differentiated from the ovule epidermal cells (Kim and Triplett, 2001; Tian et al., 2022), and it is mainly composed of cellulose, ash and cuticle, and its cellulose content exceeds 90% (Huang et al., 2021). Fiber development is usually divided into four partially overlapped stages (initiation, elongation/secondary cell wall synthesis, thickening/secondary cell wall synthesis, and maturation) (Qin et al., 2007; Gao et al., 2020). During the secondary wall thickening period, a large amount of cellulose is synthesized and deposited on the cell wall, which determines the fiber strength (Gou et al., 2007). Sucrose synthase (*SuSy*) is a key enzyme involved in the conversion in the conversion of sucrose into uridine diphosphate glucose (UDPG), and UDPG is the direct precursor for cellulose synthesis (Haigler et al., 2001; Hu et al., 2007; Zhao et al., 2024; Zhu et al., 2024). The cellulose synthase (*CesA*) complexes, also known as rosettes, use UDPG to produce β-1,4-linked glucan chains (Read and Bacic, 2002; Fujii et al., 2010). Studies have shown that the expressions of cellulose synthase *GhCesA4*, *GhCesA7* and *GhCesA8* play an important role in the development of the secondary cell wall of cotton fibers (Fujii et al., 2010; Li et al., 2015; Zhang et al., 2015; Wen et al., 2022). Studies have shown that sucrose metabolism and related activities have a strong relationship with environmental signals (Chen et al., 2014). Zhang et al. (2024) shown that water stress leads to limited sucrose hydrolysis by reducing the activity of sucrose synthase (*SuSy*), thereby increasing the sucrose content of anthers and reducing pollen fertility. Under abiotic stress conditions, increasing the amount of available sucrose in cotton fibers and leaves can improve the final quality of cotton fibers (Haigler et al., 2007; Zhao et al., 2020).

As the outermost structure of fiber cells, the wax content of the fiber epidermis is significantly negatively correlated with fiber length, strength and uniformity, and it will be significantly induced by water stress (Pan et al., 2010; Thompson et al., 2017). Studies have shown that under water stress conditions, plants increase the relative abundance of ultra-long chain alkane by changing the overall composition profile of wax, and thus significantly improve their drought resistance (Oosterhuis et al., 1991; Bondada et al., 1996). Lu et al. (2021) showed that drought stress could significantly increase the total wax content in cotton leaves, and silencing the wax synthesis-related gene *GhFAR3.1* could significantly reduce wax accumulation in leaves, thereby reducing water retention and drought tolerance. However, the response of the cotton fiber epidermis wax to water stress remains incompletely explored. In particular, the relationship between changes in wax accumulation on the fiber epidermis and quality indicators such as fiber strength remains unclear. To address these knowledge gaps, an in-depth study of the changes of fiber epidermal wax under drought conditions and its intrinsic connection with fiber quality will be helpful for the improvement and enhancement of fiber quality in dryland cotton.

In addition, genotype is a significant factor in determining the wax content of fiber epidermis (Jenks and Gore., 2017; Tomasi et al., 2021; Birrer et al., 2021; Chen et al., 2023). Previous reports have found that the wax content of green fibers is 2 to 3 times that of white cotton fibers (Pan et al., 2010). Therefore, our selected near-allelic green and white upland cotton as experimental materials, and it was hypothesized that water stress (i) would affect the supply of energy and carbon sources during cellulose synthesis, and (ii) activate the drought resistance mechanism of plants, increase the wax content on the epidermis of cotton fibers, and thereby affect the overall quality of the fibers. This study investigates the following objectives: (i) to explore the effects of water stress on the dry matter accumulation rate and fiber biomass of cotton fibers; (ii) Clarify the effects of water stress on the synthesis of sucrose and cellulose in fibroblasts and its relationship with fiber quality; (iii) Explore how the change in the wax content of the fiber epidermis affects the quality of the fiber. The results will broaden our understanding of fiber quality decline under drought conditions, and provide new ideas for cotton drought-resistance breeding and fiber quality improvement.

## 2 Materials and methods

### 2.1 Experimental design and materials

**Figure 1:**
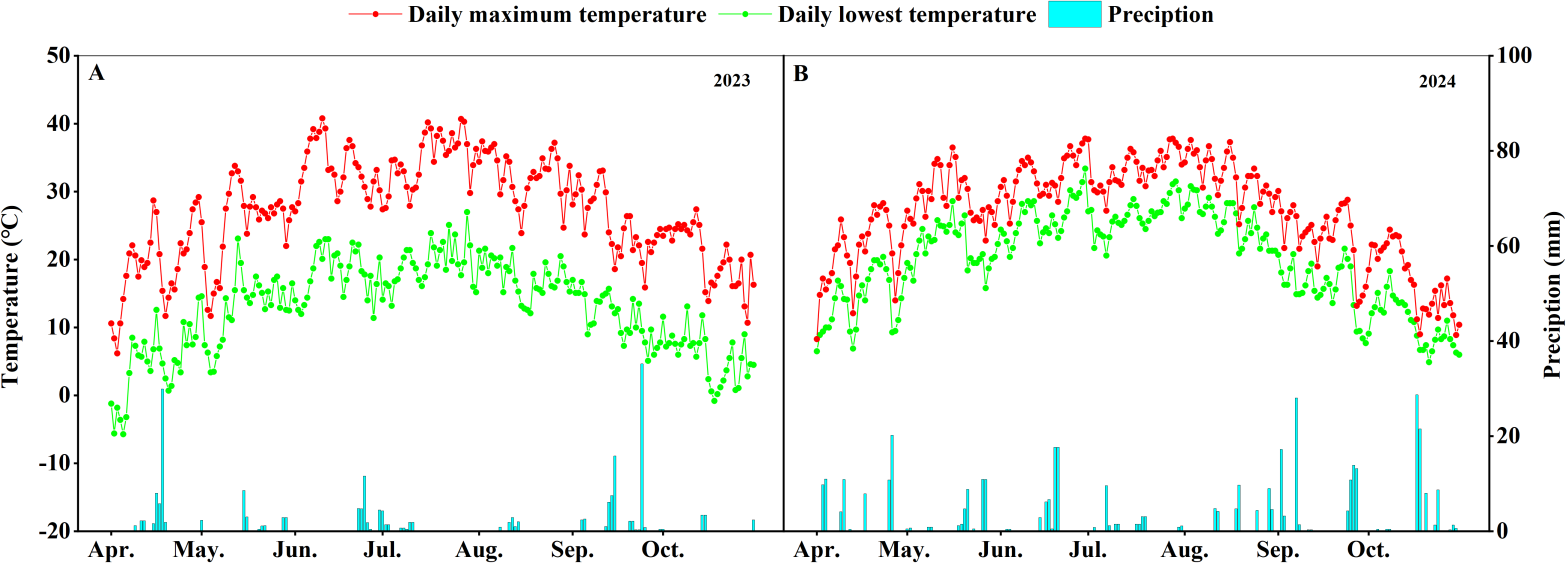
Daily maximum/minimum temperature and rainfall during the cotton growing period (2023-2024). **(A)** Maximum and minimum daily temperature and rainfall from April to September, 2023. **(B)** Maximum and minimum daily temperature and rainfall from April to September, 2024.

The field experiment was conducted at the Cotton Institute of Shihezi University (45°32′ N, 86°05′ E), during the growing seasons of 2023-2024. The soil texture in the field is loam, containing 7.33 mg/kg organic carbon, 1.32 g/kg total nitrogen, 179 mg/kg available potassium, and 7.03 mg/kg available phosphorus. The average temperature of the cotton growing season was 19.7°C and the rainfall was 211.6 mm in 2023, while the average temperature was 20.1°C and the rainfall was 401.2 mm in 2024 (**Figure 1**).

This experiment was designed as a randomized block design. The cotton cultivar Xincaimian 7 (XC7), characterized by a high wax content on the fiber epidermis, was selected as the high-wax material, while its near-isogenic line Shidamian 217 (SD217) served as the low-wax counterpart (Sun et al., 2019; **Supplementary Figure S1**). Two irrigation regimes were applied: well-watered (WW) and water-deficit (WD), each with three independent biological replicates. WW represents the local traditional irrigation scheme, and the irrigation amount of WD is 30% of that of WW (Han et al., 2025). Each subplot measured 36.8 m² (4.6 m × 8 m) with an alternating row-spacing pattern of 66 cm. This arrangement resulted in a theoretical planting density of 2.6×10⁵ plants/ha. The plots were irrigated with a surface drip-irrigation system under transparent plastic film according to published protocol (Yang et al., 2015). The irrigation treatments were applied after seedling emergence with 9 times of irrigations during the whole growth period. The irrigation amount was controlled by water meter and ball valve. In 2023 the total irrigation amount of WW and WD treatments was 4505 m^3^/ha and 1514 m^3^/ha, respectively. In 2024, the total amount of applied water was 4460 m^3^/ha and 1494 m^3^/ha, respectively (**Table 1**).

**Table 1:** Irrigation date and amount applied in the two years’ experiments (2023 and 2024).

Year	Treatment	Total irrigation volume (m^3^/ha)	Irrigation date							
			Sprout water	Jun.5	Jun.15	Jun.25	Jul.5	Jul.18	Aug.2	Aug.16
2023	WW	4505	225	700	620	620	620	620	620	480
	WD	1514	230	210	186	186	186	186	186	144
Irrigation date			Sprout water	Jun.3	Jun.13	Jun.24	Jul.2	Jul.16	Aug.1	Aug.15
2024	WW	4460	230	680	600	600	610	620	620	500
	WD	1494	225	204	180	180	183	186	186	150

WW, well-watered; WD, water-deficit.

### 2.2 Experimental sample collection

The white flowers at the first fruit node of the 3rd - 6th fruit branches were tagged on the day of flowering during the blooming stage (July 5-9, 2023 and July 1-5, 2024). Boll samples were taken every 5 days from 20 days-post-anthesis (DPA) until boll maturity (naturally open). Randomly select 6–8 cotton bolls from the same fruit branches at the same growth stage for each sampling.

### 2.3 Determination of growth parameters, fiber yield and quality

At harvest, ten representative cotton plants with comparable growth were selected from each experimental to measure the plant height, the number of fruiting branch nodes, and the number of bolls that could be harvested. Calculate the yield per unit area according to the actual harvested quantity of each plot. Three 100 g samples of cotton wool were randomly selected from each plot and sent to the China Cotton Quality Testing Center for determination of fiber quality, including elongation, uniformity, micronaire value, length and strength.

### 2.4 Fiber biomass accumulation determination

At 20, 25, 30, 40, and 50 DPA, fibers from each sampled boll were cut and dried at 40°C. The biomass of the fiber was then recorded. A logistic equation was used to model the dynamic changes in fiber biomass accumulation within the fiber, where t (d) means DPA, and Y is the fiber biomass at t. K is the theoretical maximum of fiber biomass and a and b are parameters which can be derived during the logistic regression, and can further be used for the calculation of t_1_ and t_2._


(1) $$Y=\frac{K}{1+ae^{bt}}$$

The following equation can be obtained by applying calculus to Equation 1:


(2) $${\text{t}}_{1}=\frac{1}{b}\text{ ln}\left(\frac{2+\sqrt{3}}{a}\right)$$

(3) $${\text{t}}_{2}=\frac{1}{b}\text{ln}(\frac{2-\sqrt{3}}{a})$$

(4) $$\Delta t=t_{2}-t_{1}$$

Where t_1_ is the start time of rapid increase of fiber biomass, and t_2_ is the terminate time of rapid increase of fiber biomass, so Δt is the duration of rapid increase of fiber biomass.

During T, the average increase rate of fiber biomass (V_T_) can be calculated according to formula (5) as follows:


(5) $${\text{ V}}_{T}=\frac{\Delta Y}{\Delta t}=\frac{y_{2}-y_{1}}{t_{2}-t_{1}}\;$$

The fastest speed (V_M_) of fiber biomass accumulation was calculated with the following Equation 6:


(6) $${\text{V}}_{M}\;=\frac{-bk}{4}$$

### 2.5 Cell morphology observation

Following the method described by Wang et al. (2022), the fiber sections of SD217 and XC7 plants were observed by Scanning electron microscope SU8010 (SEM). Here, fiber tissues at 30 DPA and mature cotton fibers were fixed in FAA solution (3.7% formalin, 50% alcohol and 5% glacial acetic acid) and dehydrated in graded ethanol. Fiber midsections of about 1 cm long were then embedded in resin SPI812 and cut into 8-μm-thick sections using a microtome LEICA EMUC7. Sections were observed under electron microscope SU8010. For each sample, select three slices for observation. Cell wall thickness was measured by ImageJ software.

### 2.6 Cellulose and sucrose content determination

Cellulose content of developing cotton fibers was determined using the anthrone method (Abidi et al., 2010). 0.2 g dried fiber was immersed into the acetic-nitric acid reagent for digestion, then distilled water was used to wash the samples. After drying at room temperature, the samples were dissolved in 67% H_2_SO_4_ and 0.2% anthrone reagent was added for reaction. The absorbance of the green color of the solution was measured using a UV-Vis spectrophotometer (LAMDA650, PerkinElmer, USA) at 625 nm to calculate the cellulose content.

### 2.7 Sucrose content determination

Sucrose was extracted and quantified by a modified method of Gao et al. (2020). About 0.3 g dry weight fiber samples were extracted with three successive 5 mL washes of 80% ethanol. The ethanol samples were incubated in an 80°C water bath for 30 min. Then the samples were centrifuged at 10,000×g for 10 min, and collect three equal portions of the supernatant together and measure the absorbance value at 480 nm, which is used for the measurement of sucrose. The sucrose assay was conducted according to Loka and Oosterhuis (2016).

### 2.8 Extractions and analysis of cuticular waxes on cotton fiber

The 1.5-2.0 g cotton fiber samples, after removing impurities, were placed in a -50°C freeze drying instrument and were freeze-dried for 12 h. After the fiber samples were taken out and their temperature returned to room temperature (25°C), they were weighed on an analytical balance and recorded as the fiber weight M_1_ before wax extraction. The fiber placed in 200 mL glass barrels with zeolite, and immersed in petroleum ether (boiling point 60°C). The extraction was carried out in a Soxhlet extractor. After 40 minutes of extraction, the fiber samples were taken out and placed in a freeze dryer at -50°C for 12 hours. Take out the samples and record the fiber mass M_2_ after wax extraction. The wax content in the fiber is equal to the original fiber mass minus the fiber mass after wax extraction. The cotton fiber wax content for each sample was calculated as the average wax content for the three repeated results.

### 2.9 qRT-PCR analysis

Total RNA of the fiber was extracted using RNA-prep Pure Plant Kit (Polysaccharides & Polyphenolics-Rich) (Vazyme, Nanjing, China). Then total RNA of 1 μg was used to produce cDNA with Prime Script RT Master Mix (Vazyme, Nanjing, China). The transcript levels of genes were analyzed by qRT­PCR using the Light Cycler^®^ 480 II (Roche). Each reaction was performed in 10 µL volume using SYBR Green Master Mix (Takara) under the following PCR conditions: 94°C for 1 min followed by 40 cycles of 94°C for 15 s, 59°C for 15 s, and 72°C for 20 s. The cotton poly­ubiquitin gene *GhUBQ7* was used as an internal control. The relative expression levels of target genes were calculated with the 2^−ΔΔCt^ method (Livak and Schmittgen, 2001). All primers used in this study are listed in (**Supplementary Table 1**).

### 2.10 Statistical analysis

Microsoft Excel 2019 was used to process data and Origin 2021. SPSS 19.0 was used to perform analysis of variance. Means were separated using Duncan’s multiple range test at (*p< 0.01*). Perform correlation analysis using the Pearson’s method (*P<0.01*).

## 3 Results

### 3.1 Effects of WD irrigation on cotton yield and fiber quality

Irrigation amount had significant effect on fiber uniformity, fiber strength, fiber length and fiber elongation (*P<0.01*). Compared with the WW treatment, under the WD treatment, the fiber strength, fiber length, and fiber elongation of SD217 were significantly lower by 11.66%, 9.74%, and 25.79% respectively in 2023, and significantly lower by 15.73%, 10.57%, and 19.30% respectively in 2024 (*P<0.01*) (**Table 2**). Compared with WW irrigation, under WD irrigation conditions, the seed cotton yield of SD217 decreased significantly by 70.90% and 67.39% respectively in 2023 and 2024. Similarly, the fiber strength, fiber length, and fiber elongation of the XC7 are significantly reduced under the WD treatment. In 2023, the corresponding values decreased by 15.73%, 10.57%, and 19.30%, respectively. In 2024, compared with the WW treatment, the reductions were 18.63%, 7.16%, and 15.01% respectively (*P<0.01*) (**Table 2**). At the same time, the seed cotton yield under the WD treatment decreased significantly. It decreased by 62.93% in 2023 and 60.22% in 2024 compared with the WW treatment. In conclusion, WD irrigation has a significant impact on the fiber yield and quality, resulting in a decrease in fiber strength, fiber length, and fiber elongation. It has a greater impact, in particular, on the fiber strength of XC7 and the fiber elongation of SD217.

**Table 2:** Effect of WD treatments on cotton fiber quality in 2023 and 2024.

Variety	Irrigation	Fiber uniformity (%)		Fiber strength(cN/tex)		Micronaire		Fiber length (mm)		Fiber elongation (%)		Seed cotton yield (kg/ha)	
		2023	2024	2023	2024	2023	2024	2023	2024	2023	2024	2023	2024
SD217	WW	82.80 ± 0.57a	84.37 ± 1.15a	33.22 ± 0.05a	33.87 ± 0.55a	4.77 ± 0.17a	4.89 ± 0.04a	28.14 ± 0.21a	29.25 ± 0.22a	5.70 ± 0.29a	5.23 ± 0.21a	8169.94 ± 74.74a	8258.37 ± 65.1a
	WD	81.93 ± 0.25b	83.90 ± 0.5b	29.35 ± 0.16b	30.67 ± 1.01b	4.86 ± 0.04a	4.89 ± 0.02a	25.40 ± 0.17c	27.88 ± 0.47b	4.23 ± 0.56b	3.57 ± 0.15b	2377.36 ± 108.17c	2693.34 ± 97.87c
XC7	WW	82.33 ± 0.66ab	82.37 ± 0.46c	21.11 ± 0.45c	21.47 ± 0.42c	2.48 ± 0.05b	3.08 ± 0.17b	27.25 ± 0.15b	27.39 ± 0.45b	4.30 ± 0.22b	3.73 ± 0.06b	4909.89 ± 69.93b	5074.8 ± 108.81b
	WD	80.27 ± 0.21c	80.47 ± 0.5d	17.79 ± 0.34d	17.47 ± 0.47d	2.67 ± 0.13b	2.68 ± 0.06c	24.37 ± 0.29d	25.43 ± 0.75c	3.47 ± 0.21c	3.17 ± 0.23c	1820.05 ± 59.51d	2018.89 ± 71.4d
Source of variance													
Variety		<0.05	<0.01	<0.01	<0.01	<0.01	<0.01	<0.01	<0.01	<0.01	<0.01	<0.01	<0.01
Irrigation		<0.01	<0.05	<0.01	<0.01	0.108	<0.01	<0.01	<0.01	<0.01	<0.01	<0.01	<0.01
Variety ×Irrigation×Years		NS		NS		NS		NS		NS		NS	

WW, well-watered; WD, water-deficit; SD217, Shidamian 217; XC7, Xincaimian 7. Means with different letters indicate significant different at *p< 0.05.* NS indicates no significance at *p< 0.05*.

### 3.2 Effects of WD irrigation on fiber biomass accumulation

**Figure 2:**
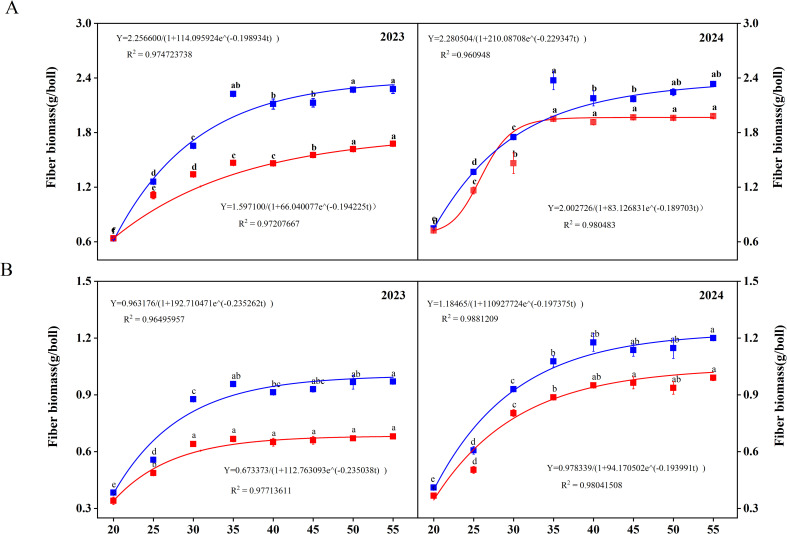
The dynamic changes of dry matter accumulation during the development process of cotton fibers (2023-2024). **(A)** The dynamic changes of dry matter accumulation during the development process of cotton fibers of SD212. **(B)** The dynamic changes of dry matter accumulation during the development process of cotton fibers of XC7 SD217, Shidamian 217. XC7, Xincaimian 7. DPA, days-post-anthesis. WW, well-watered; WD, water-deficit. Values represent the means ± SD. Different letters on top of the bars indicate significant difference at *P< 0.05*.

WD treatment significantly inhibited the accumulation of biomass (Supplemental **Supplementary Figure S2**). Under WW irrigation, SD217 exhibited significant accumulation in the biomass of fiber during 20–35 DPA, especially within 20–30 DPA (**Figure 2A**). For XC7, the biomass of fiber increased significantly within 20–30 DPA (**Figure 2B**). Under WD irrigation, the biomass accumulation rate of SD217 and XC7 significantly decreased after 30 DPA. In WD irrigation, the fiber biomass accumulation of SD217 and XC7 decreased by 23.62% and 30.82% respectively, relative to that in WW irrigation (**Figures 2A,B**). The fiber biomass accumulation was modeled using the logistic growth equation (Equation 1), as shown in **Figure 2**. Accordingly, the growth rate of fiber was calculated according to the Equations 2–6. For SD217 variety, it showed that the fast accumulation period (FAP) of fiber biomass was 13 days (17–30 DPA) under WW irrigation (**Table 3**). The average rate of growth rate was 0.10 g^-2^/d, with the highest rate being 0.12 g^-2^/d. Similarly, for XC7 variety, the FAP of fiber biomass was 11 days (17–28 DPA), and the average growth rate was 0.05 g^-2^/d, with the highest growth rate being 0.06 g^-2^/d. For both varieties, the FAP began 2 days earlier under conditions other than WW irrigation, and the average growth rate of SD217 and XC7 decreased by 29.00% and 24.40%, respectively (**Table 3**). However, WD treatment severely restricted the photosynthetic rate and photosynthate output of the subtending leaves, which may be one of the main reasons for the decrease in the dry matter accumulation rate of cotton.

**Table 3:** Effects of irrigation levels on biomass accumulation of fiber biomass in different varieties (2023 and 2024).

Year	Treatments	R^2^	t_1_ (DPA)	t_2_ (DPA)	T/d	V_T_ (g^−2^ d^−1^)	V_M_ (g^3−2^ d^−1^)
2023	WW-SD217	0.9747	17.192	30.432	13.240	0.098	0.112
	WD-SD217	0.9721	14.794	28.355	13.561	0.068	0.078
	WW-XC7	0.9650	16.765	27.961	11.196	0.050	0.057
	WD-XC7	0.9771	14.501	25.708	11.206	0.035	0.040
2024	WW-SD217	0.9776	18.193	30.123	11.930	0.122	0.139
	WD-SD217	0.9727	14.868	28.301	13.433	0.077	0.088
	WW-XC7	0.9649	16.824	27.991	11.167	0.056	0.064
	WD-XC7	0.9804	14.597	25.664	11.067	0.039	0.045

WW, well-watered; WD, water-deficit; SD217, Shidamian 217; XC7, Xincaimian 7. DPA indicates days-post-anthesis (d). t_1_ and t_2_ are the beginning and terminating date of the fast accumulation period (FAP). T indicates the duration of the FAP (T = t_2_-t_1_). V_M_ and V_T_ are the maximum and average rates during the FAP, respectively.

### 3.3 Morphological observation of fiber cell wall

**Figure 3:**
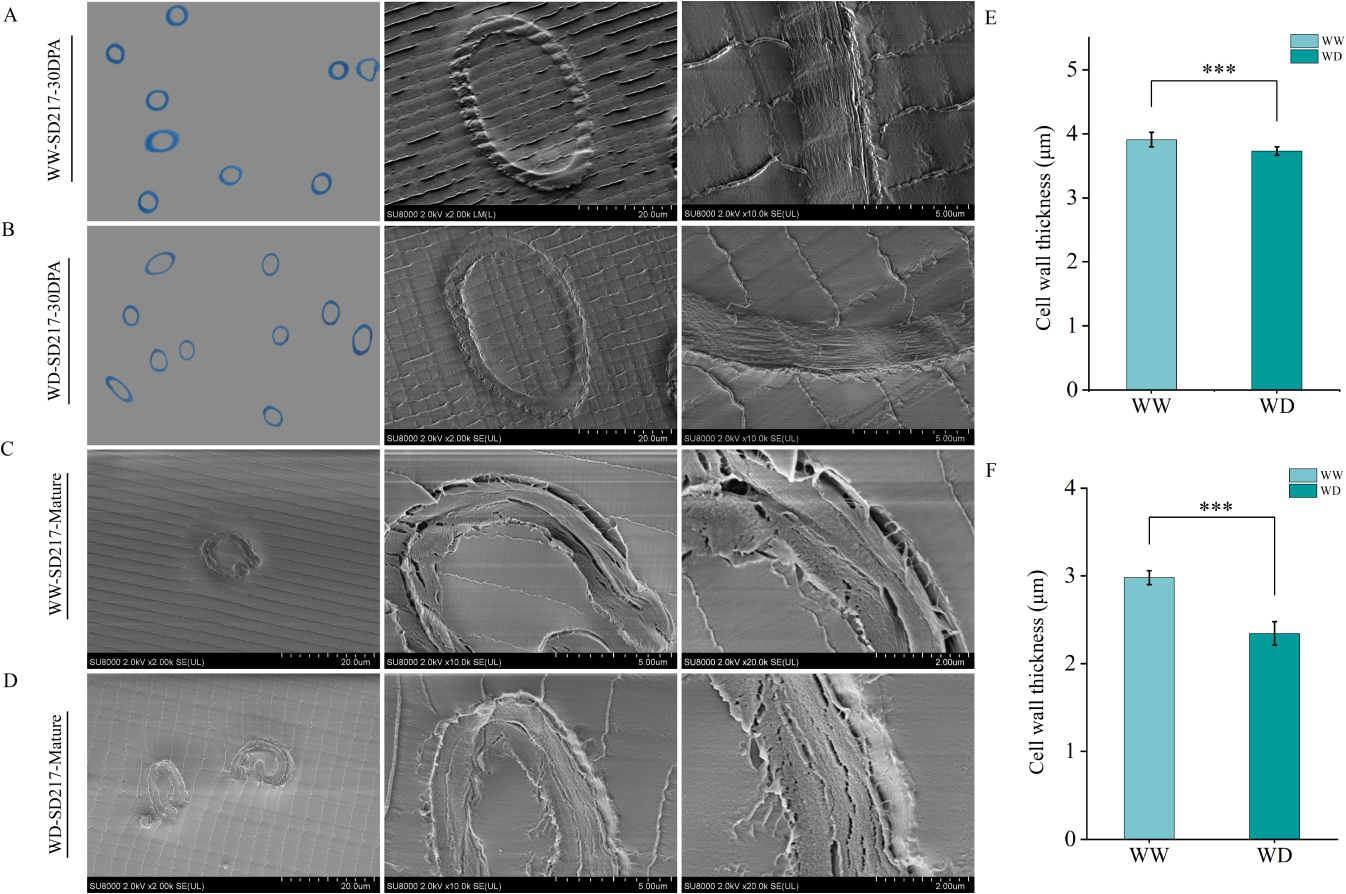
The influence of WD treatment on the cell wall thickness of SD217 fibers (2024). **(A)** Scanning Electron Microscope image of the thickness of the fiber cell wall of SD217 at 25 DPA under the WW treatment. **(B)** Transmission electron microscope image of the thickness of the fiber cell wall of SD217 at 25 DPA under the WD treatment. **(C)** Transmission electron microscope image of the thickness of the fiber cell wall of SD217 at the mature stage under the WW treatment. **(D)** Transmission electron microscope image of the thickness of the fiber cell wall of SD217 at the mature stage under the WD treatment. **(E)** Quantitative comparison of the thickness of the fiber cell wall of SD217 at 25 DPA under the WW and WD treatments. **(F)** Quantitative comparison of the thickness of the fiber cell wall of SD217 at the mature stage under the WW and WD treatments. SD217, Shidamian217. DPA, days-post-anthesis. WW, well-watered; WD, water-deficit. Values represent the means ± SD. * *p*<=0.05 ** *p*<=0.01 *** *p*<=0.001.

**Figure 4:**
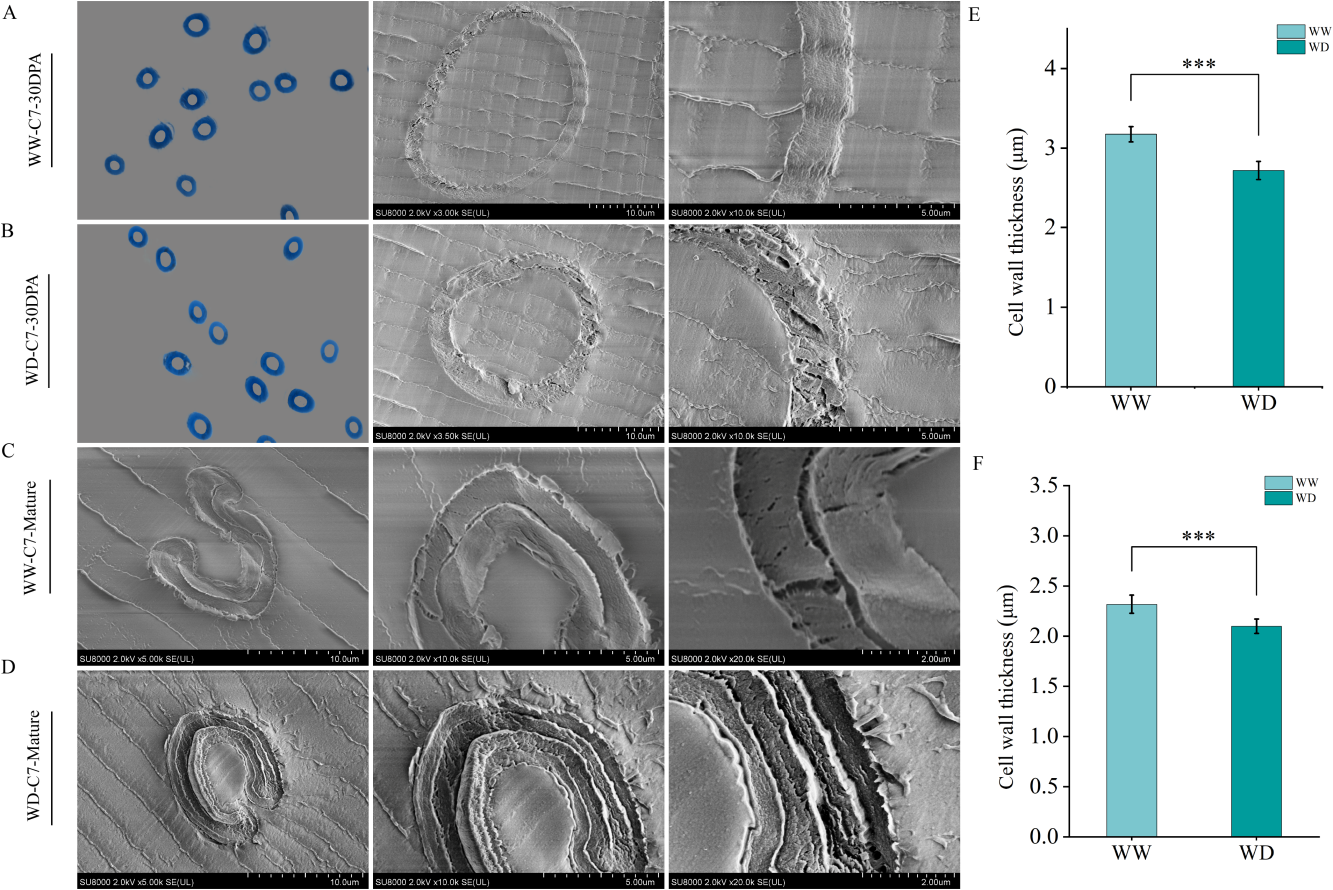
The influence of WD treatment on the cell wall thickness of XC7 fibers (2024). **(A)** Scanning Electron Microscope image of the thickness of the fiber cell wall of XC7 at 25 DPA under the WW treatment. **(B)** Transmission electron microscope image of the thickness of the fiber cell wall of XC7 at 25 DPA under the WD treatment. **(C)** Transmission electron microscope image of the thickness of the fiber cell wall of XC7 at the mature stage under the WW treatment. **(D)** Transmission electron microscope image of the thickness of the fiber cell wall of XC7 at the mature stage under the WD treatment. **(E)** Quantitative comparison of the thickness of the fiber cell wall of XC7 at 25 DPA under the WW and WD treatments. **(F)** Quantitative comparison of the thickness of the fiber cell wall of XC7 at the mature stage under the WW and WD treatments. XC7, Xincaimian 7. DPA, days-post-anthesis. WW, well-watered; WD, water-deficit. Values represent the means ± SD. * *p*<=0.05 ** *p*<=0.01 *** *p*<=0.001.

In order to further analyze the effect of WD treatment on fiber quality, resin embedding was performed on 30 DPA and mature fiber, and morphological characteristics of fiber was observed by SEM (**Figures 3A–D**, **4A–D**). In this study, we found that WD irrigation significantly reduced fiber cell wall thickness. The fiber cell wall thickness at 30 DPA was decreased by 10.59% and 9.50%, respectively (**Figures 3E**, **4E**), and the fiber cell wall thickness at mature stage was decreased by 12.66% and 14.31%, respectively (**Figures 3F**, **4F**). When comparing the effects of WD irrigation and WW treatment, we observed that the fiber cell wall accumulated thicker cuticle wax under WD irrigation conditions. WD irrigation makes the fiber cell wall thinner and the cuticular wax layer thicker. These morphological changes may lead to a decrease in fiber strength and elongation. The thinner cell wall weakens the structural support, while the thicker wax layer reduces the flexibility and extensibility of the fibers. Overall, the morphological changes caused by WD irrigation significantly affect the fiber properties, indicating a close relationship between the fiber structure and its function.

### 3.4 Determination of cellulose content

**Figure 5:**
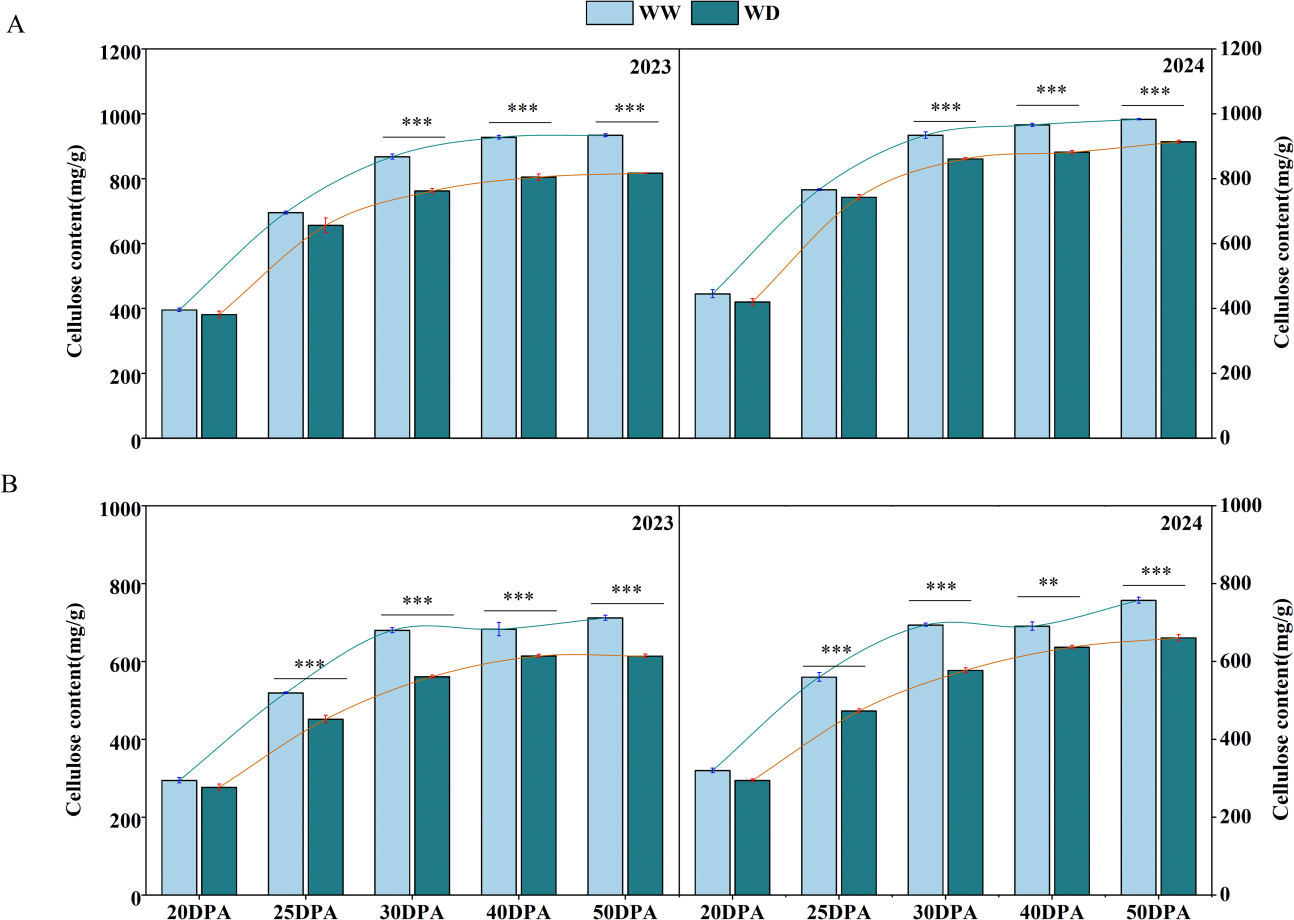
The influence of WD treatment on the contents of cellulose in cotton fibers at various stages of fiber development (2023-2024). **(A)** The cellulose content in the fibers of SD217 at various stages of fiber development. **(B)** The cellulose content in the fibers of XC7 at various stages of fiber development. SD217, Shidamian 217. XC7, Xincaimian 7. DPA, days-post-anthesis. WW, well-watered; WD, water-deficit. Values represent the means ± SD. * *p*<=0.05 ** *p*<=0.01 *** *p*<=0.001.

The deposition of cellulose determines the thickness of the secondary cell wall of fiber, and mature cotton fiber contains more than 90% crystalline cellulose (Huang et al., 2021). In order to investigate the mechanism of cell wall thickness variation, the content of cellulose was measured. During the fiber development, the cellulose content showed a trend of gradual increase. The cellulose content increased sharply during the 20–30 DPA. During this time, the cellulose accumulation rates of SD217 and XC7 were 48.06 mg/d and 37.99 mg/d respectively. In contrast, the change in cellulose content tended to stabilize during the 40–50 DPA (**Figure 5**). At 30 DPA, the cellulose content of SD217 reached 900.67 mg/g, and that of XC7 was 686.66 mg/g. WD irrigation limited the accumulation of cellulose content in each period. Compared with WW irrigation, the cellulose content of SD217 at 30 DPA decreased significantly by 9.91% (*p<0.01*) under WD irrigation. Compared with WW irrigation at 25 DPA and 30 DPA, the cellulose content of XC7 was significantly reduced by 14.35% and 17.17% (*p<0.01*). In the mature stage, the cellulose content of SD217 and XC7 was significantly reduced by 9.70% and 13.23% (*p<0.01*) under WD irrigation, respectively, compared with those under WW irrigation (**Figure 5**). To conclude, at 20–30 DPA, the accumulation of cellulose is crucial for the development of cotton fibers. The rapid accumulation of cellulose at this stage is key to the formation and strengthening of the cell wall. However, the WD irrigation treatment inhibits the cellulose accumulation process in cotton fibers, resulting in a decrease in cell wall thickness and ultimately having a negative impact on the quality of cotton fibers.

### 3.5 Determination of sucrose content

**Figure 6:**
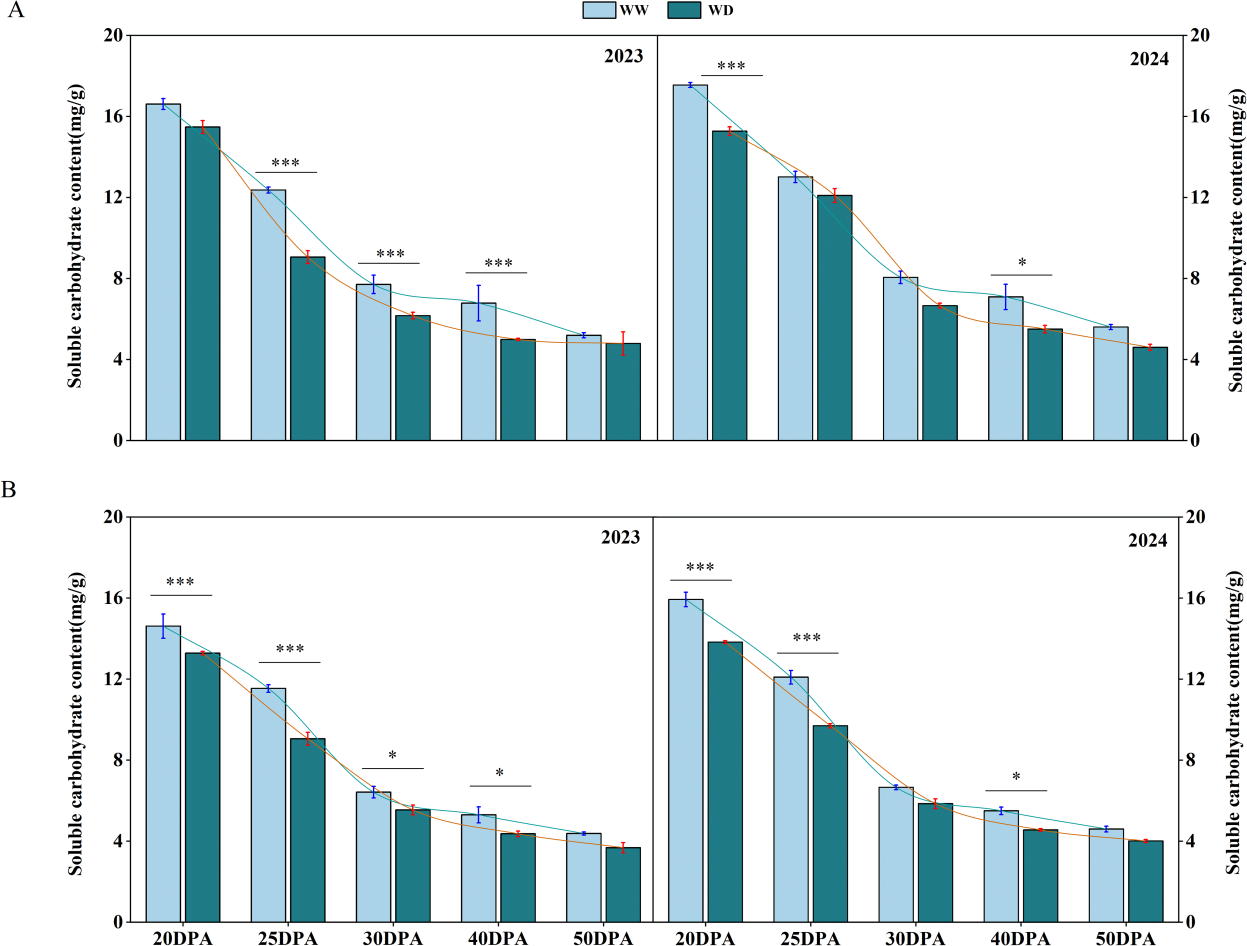
The influence of WD treatment on the contents of sucrose in cotton fibers at various stages of fiber development (2023-2024). **(A)** The sucrose content in the fibers of SD217 at various stages of fiber development. **(B)** The sucrose content in the fibers of XC7 at various stages of fiber development. SD217, Shidamian 217. XC7, Xincaimian 7. DPA, days-post-anthesis. WW, well-watered; WD, water-deficit. Values represent the means ± SD. * *p*<=0.05 ** *p*<=0.01 *** *p*<=0.001.

With the development of cotton fiber, the sucrose content in cotton fiber showed a trend of high in the early stage and low in the late stage. The sucrose contents of SD217 and XC7 fiber were 17.08 mg/g and 15.27 mg/g during the 20 DPA, and 5.40 mg/g and 4.49 mg/g in the 50 DPA, respectively (**Figure 6**). WD irrigation had a certain inhibitory effect on the sucrose content of fiber in 20–40 DPA. Under WD irrigation, the sucrose content of SD217 at 25, 30 and 40 DPA of fiber development was significantly reduced by 16.91%, 18.66% and 24.42%, respectively, compared with WW irrigation. Similarly, the sucrose content of XC7 at 25, 30 and 40 DPA during fiber development was also significantly reduced by 20.67%, 12.85% and 17.41%, respectively, compared with WW irrigation (**Figure 6**). When the fiber developed to 50 DPA, the sucrose content in the fiber was less affected by water stress. There was no significant difference in fiber sucrose content between the two varieties under WD irrigation and WW irrigation. In summary, WD irrigation reduced the content of sucrose in each period of fiber development. Among them, the effect on fiber development of 20–40 DPA (secondary wall thickening) was more serious, and there was no significant effect on fiber development after 50 DPA. In conclusion, the WD treatment reduces the sucrose content in cotton fibers, which implies that during the fiber development process, there is an insufficient supply of energy and carbon sources for cellulose synthesis. Therefore, it may slow down the accumulation of cellulose, weaken the formation and strengthening of the cell wall, and ultimately lead to a decline in fiber quality.

### 3.6 WD irrigation effects on fiber wax

**Figure 7:**
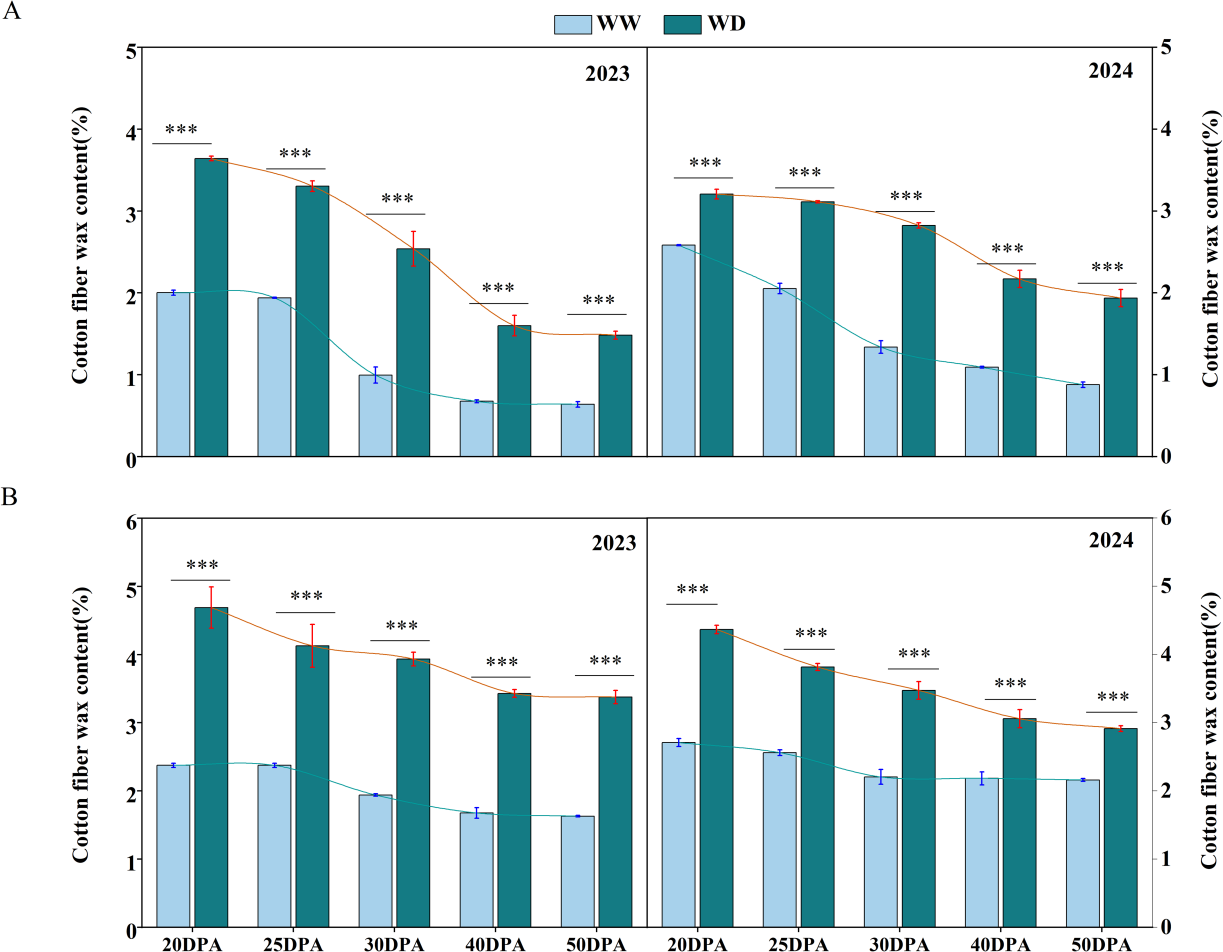
The influence of WD treatment on the contents of wax in cotton fibers at various stages of fiber development (2023-2024). **(A)** The wax content in the fibers of SD217 at various stages of fiber development. **(B)** The wax content in the fibers of XC7 at various stages of fiber development. SD217, Shidamian 217. XC7, Xincaimian 7. DPA, days-post-anthesis. WW, well-watered; WD, water-deficit. Values represent the means ± SD. * *p*<=0.05 ** *p*<=0.01 *** *p*<=0.001.

By measuring the wax content of fiber epidermis at different development stages of cotton fiber, it was found that the wax content decreased gradually with the development of cotton fiber. At the 20 DPA, the fiber epidermal wax content of SD217 and XC7 was relatively high, accounting for 2.29% and 2.54% of the total dry weight, respectively (**Figure 7**). After entering 40 DPA, the wax content of cotton fiber tended to be stable, and the wax content of fiber epidermis of SD217 and XC7 was 0.88% and 1.93%, respectively (**Figure 7**). In addition, the wax content in each stage was significantly increased by water stress. Compared with WW treatment, under WD conditions, the fiber epidermal wax content of SD217 and XC7 at 20DPA was significantly increased by 53.01% and 79.33%, respectively, accounting for 3.42% and 4.53% of the total dry weight. At 40 DPA, the wax content of fiber epidermis of SD217 and XC7 under water stress treatment was 1.88% and 3.24%, respectively, which was 117.86% and 72.27% higher than that of WW treatment, respectively (**Figure 7**). At 50 DPA, compared with the WW treatment, the wax content on the surface of fibers of varieties SD217 and XC7 under the WD treatment increased significantly by 126.49% and 71.08%, respectively (**Figure 7**). The results show that, in a water-stressed environment, cotton fibers increase the wax content of their epidermis as a defense mechanism. However, this thickening of the wax layer reduces the extensibility of fiber cells, thereby having a negative impact on fiber quality.

### 3.7 WD regulates the expression of genes related to cellulose and wax synthesis in cotton.

**Figure 8:**
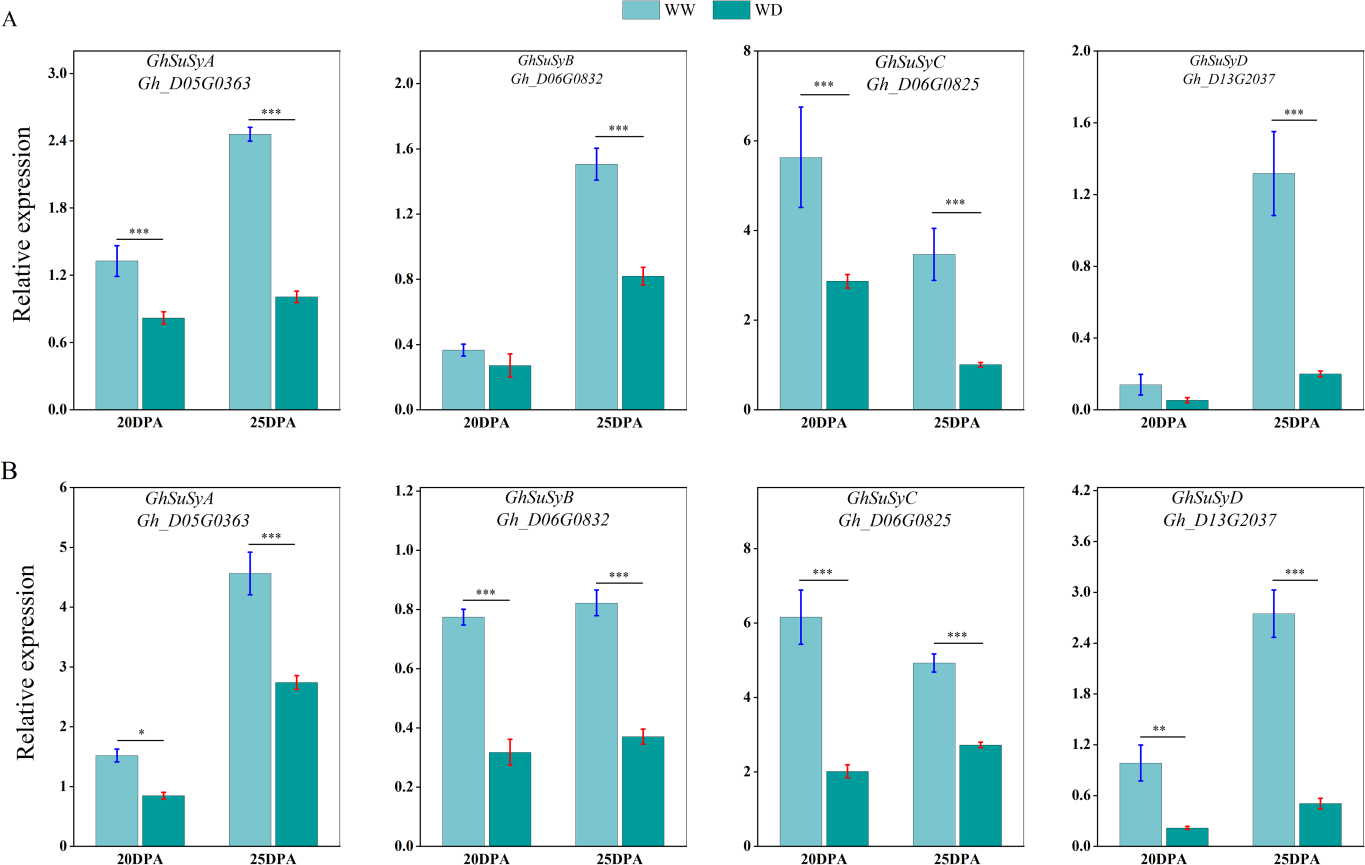
The influence of WD treatment on the expression level of sucrose synthase gene in fibers (2024). **(A)** The expression levels of the sucrose synthase gene in the fibers of SD217 at 20DPA and 25DPA under the WW and WD treatments. **(B)** The expression levels of the sucrose synthase gene in the fibers of XC7 at 20DPA and 25DPA under the WW and WD treatments. DPA, days-post-anthesis. WW, well-watered; WD, water-deficit. Values represent the means ± SD. * *p*<=0.05 ** *p*<=0.01 *** *p*<=0.001.

**Figure 9:**
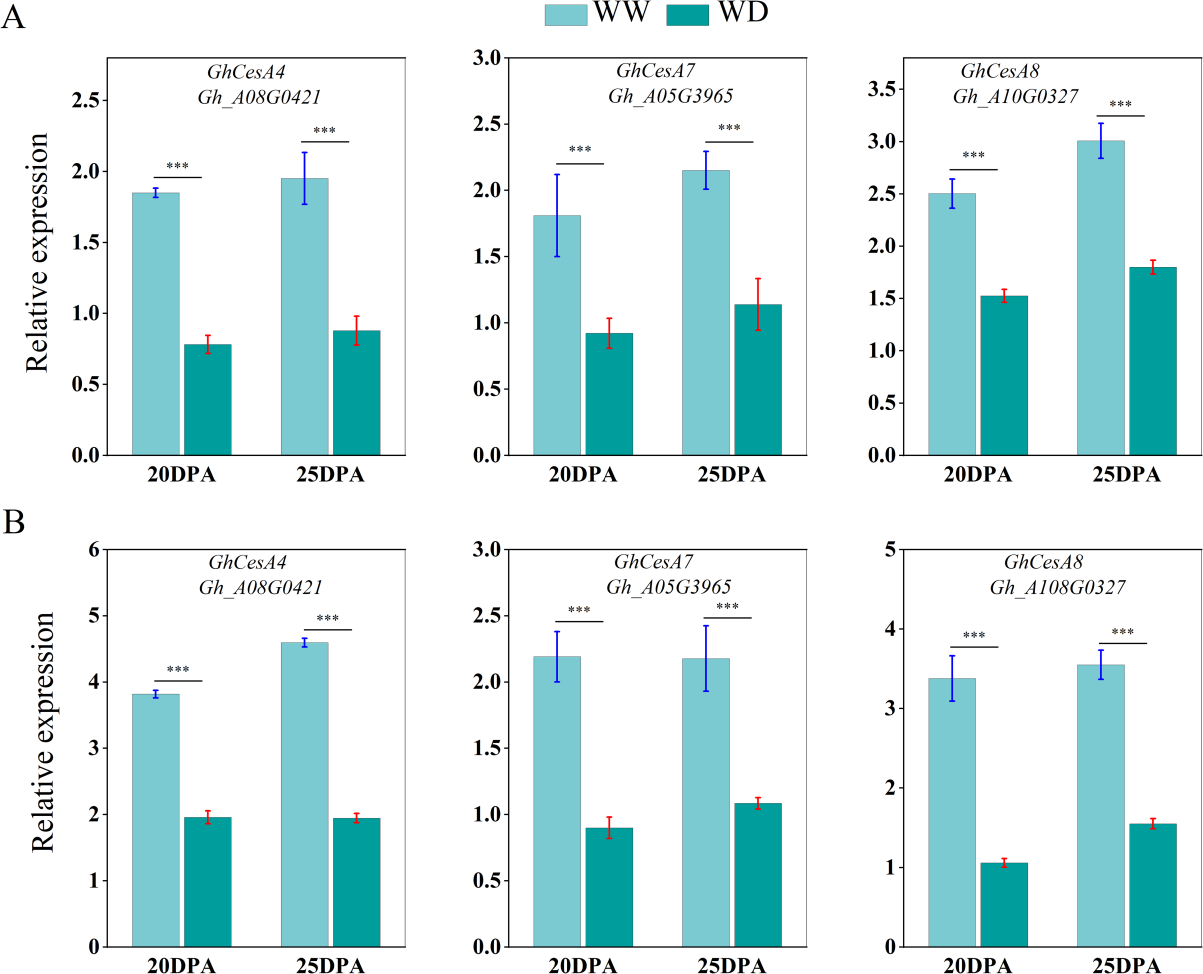
The influence of WD treatment on the expression level of cellulose synthase gene in fibers (2024). **(A)** The expression levels of the cellulose synthase gene in the fibers of SD217 at 20DPA and 25DPA under the WW and WD treatments. **(B)** The expression levels of the cellulose synthase gene in the fibers of XC7 at 20DPA and 25DPA under the WW and WD treatments. DPA, days-post-anthesis. WW, well-watered; WD, water-deficit. Values represent the means ± SD. * *p*<=0.05 ** *p*<=0.01 *** *p*<=0.001.

**Figure 10:**
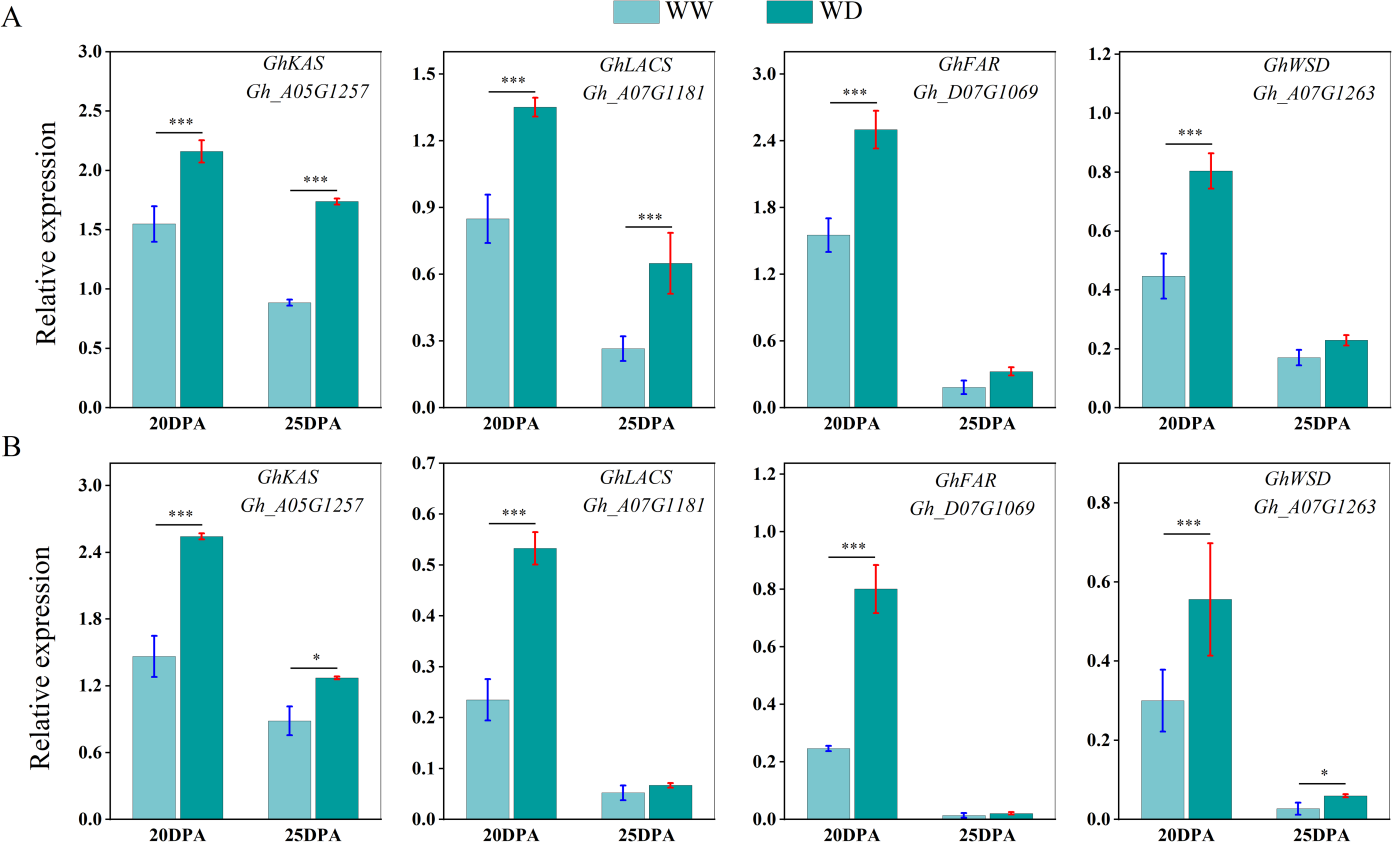
The influence of WD treatment on the expression levels of genes related to wax synthesis in fibers (2024). **(A)** The expression levels of genes related to wax synthesis in the fibers of SD217 at 20DPA and 25DPA under the WW and WD treatments. **(B)** The expression levels of genes related to wax synthesis in the fibers of XC7 at 20DPA and 25DPA under the WW and WD treatments. DPA, days-post-anthesis. WW, well-watered; WD, water-deficit. Values represent the means ± SD. * *p*<=0.05 ** *p*<=0.01 *** *p*<=0.001.

We used qRT-PCR to analyze the expression of sucrose synthase, cellulose synthase and wax synthesis-related genes in SD217 and XC7 cotton fibers during 20 and 25 DPA cellulose synthesis and accumulation under WW and WD treatments. At 20 and 25 DPA, compared with WW treatment, the expression of Sus gene in the two varieties was significantly reduced under WD treatment. At 20 DPA, the expression level of *GhSusyC* gene was most affected by WD treatment, and the relative expression levels of the gene were significantly reduced by 49.09% and 67.29%, respectively, compared with WW treatment. At 25 DPA, the expression level of *GhSuSyD* gene was significantly inhibited under WD treatment, which was significantly reduced by 84.82% and 81.62% compared with WW treatment (**Figure 8**). Cellulose synthase (*GhCesA*) is responsible for the biosynthesis of cellulose. *GhCesA4*, *GhCesA7* and *GhCesA8* are reported to play a key role in the development of fiber secondary wall. Compared with WW cotton fiber, the expression levels of cellulose synthase genes *GhCesA4*, *GhCesA7* and *GhCesA8* in SD217 and XC7 varieties were significantly decreased under WD treatment, and the decrease in XC7 was greater (**Figure 9**). Wax is a mixture of esters of long linear carboxylic acids and long linear alcohols. In the *de novo* synthesis of fatty acids, β-ketoacyl-acyl carrier protein synthetase (*KAS*) plays a crucial role in the polymerization of acyl-β-ketoacyl chains. Long-chain acyl-CoA synthetase (*LACS*) is responsible for catalyzing the formation of acyl-CoA from free fatty acids. Regarding the synthesis of waxy components, fatty acyl-CoA reductase (*FAR*) catalyzes the reduction of long-chain acyl-CoA to primary alcohols. Finally, wax esters are formed via wax ester synthase (*WSD*). Under the WD treatment, the expression levels of *GhKAS*, *GhLACS*, *GhFAR*, and *GhWSD*. were significantly induced (**Figure 10**). This phenomenon reasonably explains the reason for the decrease in cellulose content in cotton fibers under WD treatment. At the same time, as a key substance for plants to respond to water stress, wax can be induced by drought stress to upregulate the expression of genes related to wax metabolism in cotton fibers, thus significantly increasing the wax content in the fiber epidermis.

### 3.8 Correlation analysis

**Figure 11:**
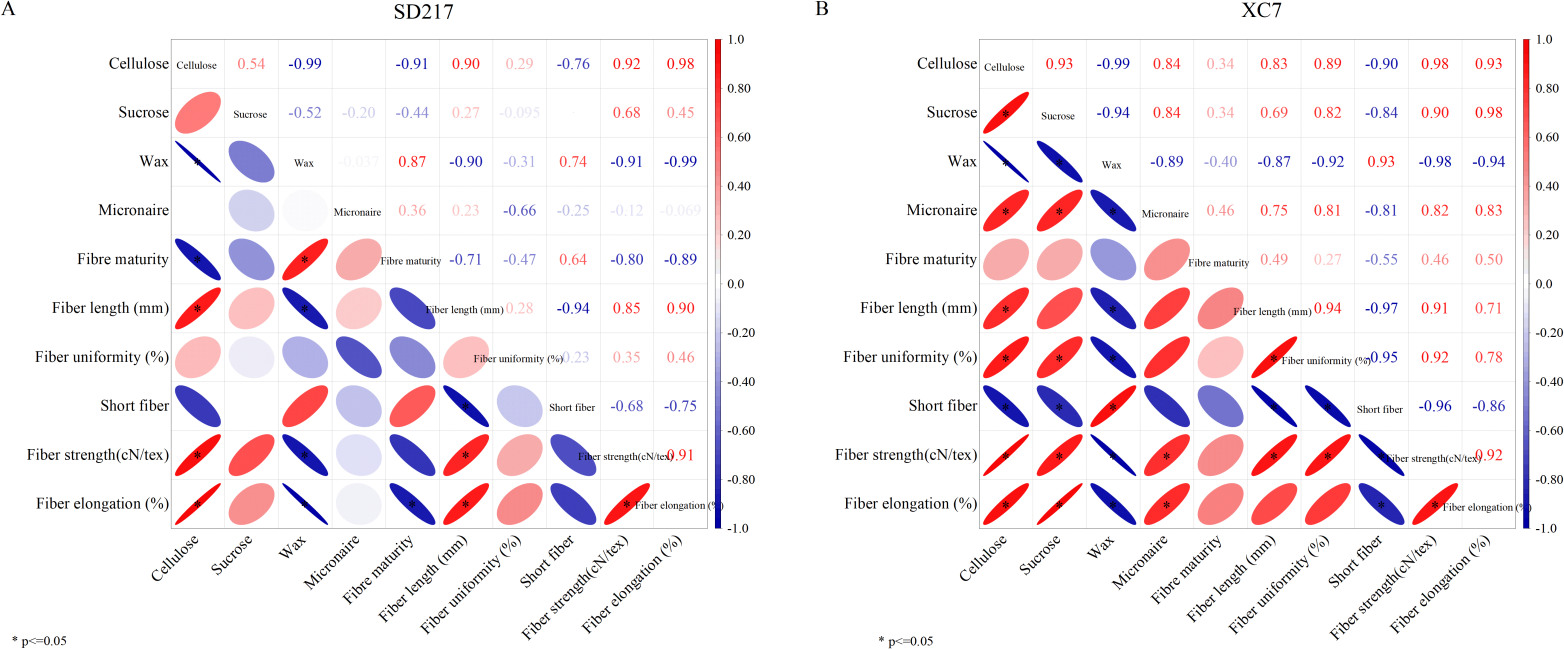
Correlation analysis between the contents of cellulose, sucrose and wax in cotton fibers and fiber quality (2023-2024). **(A)** The correlation between the contents of cellulose, sucrose and wax in the fibers of SD217 and the fiber quality. **(B)** The correlation between the contents of cellulose, sucrose and wax in the fibers of XC7 and the fiber quality. The numbers of abscissa from −1 to 1 indicate significance at the 0.05 level (both sides).

Through the correlation analysis of cellulose content, sucrose content, fiber wax content, and fiber quality indices, we found that the cellulose content of SD217 and XC7 varieties was significantly positively correlated with the sucrose content. It was significantly negatively correlated with the fiber wax content, and the correlation coefficient (r) within the 95% confidence interval was -0.99 (**Figure 11**). Meanwhile, fiber strength, fiber length, and fiber elongation were positively correlated with the cellulose content, and the correlation coefficient (r) values were 0.92, 0.90, 0.98 (for SD217) and 0.98, 0.83, 0.93 (for XC7), respectively. They were significantly negatively correlated with the fiber wax content, and the correlation coefficient (r) values were -0.99, -0.90, -0.99 (for SD217) and -0.98, -0.87, -0.94 (for XC7), respectively. Additionally, the short-fiber was significantly negatively correlated with fiber length and cellulose content, but significantly positively correlated with the wax content. Fiber length and strength were also positively correlated with the sucrose content, and this correlation was more significant in XC7 fibers. Therefore, fiber quality is not only affected by the contents of cellulose and sucrose, and there is also a direct correlation between the wax content and fiber quality.

## 4 Discussion

### 4.1 WD affects fiber quality by reducing the fiber biomass accumulation

With global warming, agricultural droughts are increasing, threatening cotton production. Many studies have shown drought harms cotton fiber quality and reduces its economic value (Abdelraheem et al., 2019; Gao et al., 2021; Hu et al., 2021). Unsurprisingly, our study found that WD irrigation had significant effect on fiber quality, leading to a decrease in fiber strength, fiber length, and fiber elongation (**Table 2**). This study further investigated the dynamic process of fiber development under water stress. It was found that, compared with the WW treatment, the WD treatment reduced the dry matter accumulation rates of the fiber biomass of SD217 and XC7 (**Figure 2**; **Table 2**). This finding is in agreement with previous studies, such as Hu et al. (2018) and Han et al. (2025) which also demonstrated that water stress affects fiber quality. Drought severely restricts the photosynthetic rate and the output of photosynthetic products in cotyledons (Zou et al., 2022). Drought inhibits the flow of photosynthetic products from the seed coat to cotton fibers, resulting in a higher proportion of photosynthetic products remaining in the seed coat (Zhu et al., 2024). This is the reason for the decline in cotton fiber biomass accumulation.

### 4.2 WD leads to the decrease in the sucrose and cellulose contents, and reduces the thickness of the fiber cell walls

Sucrose is the main photosynthetic transport product in plants and plays a crucial role in the synthesis and accumulation of cellulose (Ruan et al., 2003; Zhu et al., 2024). This study shows that water stress significantly reduces the cellulose content in cotton fibers (**Figure 5**). The reason may be that water stress interferes with the transport process of sucrose to cotton fibers, resulting in a shortage of energy and carbon source supply required for cellulose synthesis (Wang et al., 2022). This speculation has been verified in the determination results of sucrose content (**Figure 8**). Cellulose can endow the cell wall with toughness and is essential for the formation of fiber strength (Tausif et al., 2018 ; Huang et al., 2021; Zhang et al., 2024). Through SEM observation, it was found that under WD treatment condition, the cell wall thickness of cotton fibers at 30 DPA and mature fibers was significantly thinner than that under WW treatment condition (**Figures 3**, **4**). Correlation analysis shows that there is a significant positive correlation between fiber strength and the cellulose level in fibers (**Figure 11**). Therefore, the decrease in cellulose content under drought conditions can largely explain the reduction in fiber strength, and this conclusion is consistent with previous research reports (Huang et al., 2021; Xing et al., 2023; Zhu et al., 2024). We conclude that the WD treatment reduces sucrose content in cotton fibers, thereby decreasing the energy and carbon source supply necessary for cellulose synthesis and subsequently leading to a reduction in cellulose content. This cellulose depletion causes thinning of the fiber secondary cell wall thickness, which is the primary factor contributing to the decline in fiber strength. Therefore, in practical applications, targeted measures addressing key nodes in sucrose metabolism and transport could be adopted to enhance cotton fiber quality and yield.

### 4.3 WD leads to the increase of fiber wax content

Cuticle wax is the outermost structure of cotton fiber, and the content of wax in fiber epidermis has a direct effect on the quality of cotton fiber (Pan et al., 2010; Thompson et al., 2017). Our study shows that the content of epidermal wax in fibers is significantly negatively correlated with fiber length, strength, and fiber elongation rate, while it is significantly positively correlated with the proportion of short fibers, which was consistent with previous research results (Birrer et al., 2021). Water stress causes an increase in the epidermal wax content of cotton fibers (**Figure 7**). The results of scanning electron microscopy observation and correlation analysis show that, under the condition of water stress, a thicker layer of wax accumulates in the epidermal layer of the fibers, and there is a significant negative correlation between the wax content and the contents of sucrose and cellulose (**Figure 11**). According to Mundim and Elizabeth (2018), this phenomenon may be attributed to plants’ adaptive response to adversity. To ensure survival, plants reconfigure their metabolic pathways, decreasing the allocation of sucrose toward cellulose synthesis while redirecting more resources to epidermal wax production (Gao et al., 2020; Zhu et al., 2024). Therefore, when plants face adverse conditions such as water stress, they will preferentially allocate resources to the synthesis of epidermal wax to enhance their own protective capabilities. This process will lead to a reduction in the supply of sucrose for cellulose synthesis, which in turn makes the fiber cell walls thinner, while the cuticular wax layer becomes thicker. These morphological changes may cause a decrease in the strength and elongation rate of the fibers, thus having a negative impact on the fiber quality.

### 4.4 WD induced expression of relevant genes that play an important role in cellulose and wax synthesis

Cellulose synthesis is a complex biological process that relies on a series of enzymes and substrates (Pedersen et al., 2023). Sucrose, serving as the primary carbon substrate, is degraded by *SuSy* to provide UDP-glucose for cellulose synthesis (Delmer and Amor, 1995; Haigler et al., 2001; Hu et al., 2007). In this study, it was found that under drought stress, the expression of *GhSusy* in the fibers at 20–25 DPA during the early fiber development stage decreased significantly. This also corresponds to the results of the decrease in the contents of sucrose and cellulose in the fiber cells during this period (**Figures 5**, **6**). It has also been shown by research that the accumulation of sucrose (including sucrose and its hydrolysates) was increased in cottonseed coat but decreased in cotton fiber under drought (Zhu et al., 2024). Additionally, the reduction of Sus activity appears to contribute to a decrease in the consumption of sucrose in downstream anabolism (including cellulose and β-1, 3-glucan synthesis), which helps plants survive in adverse conditions and contributes to enhancing drought resistance (McDowell et al., 2008). This result further explains the reason for the decrease in the expression level of *GhSusy* under WD treatment (**Figures 8**, **9**). Gao et al. (2020) found in their study that under drought conditions, the sucrose content and sucrose synthase (*SuSy*) activity in cotton fibers decreased before 24 DPA, thereby slowing down the fiber elongation rate and ultimately affecting fiber length. Other studies have also shown that single high-temperature or drought stress can limit cellulose synthesis by reducing the activities of sucrose synthase (*SuSy*), soluble acid invertase (VIN) and alkaline invertase (CIN) involved in sucrose degradation (Hu et al., 2022). The cellulose synthase complex was sensitive to adverse environmental effects, which directly affected cellulose synthesis (Chen et al., 2014; Li et al., 2015; Zhang et al., 2021). This study shows that, compared with WW treatment, WD treatment of SD217 and XC7 significantly down-regulated the expression levels of *GhCesA4*, *GhCesA7* and *GhCesA8* in cotton fiber. This may be an important factor leading to the insufficient accumulation of cellulose in the secondary cell walls of cotton fiber cells, which was consistent with Padmalatha et al. (2012). In the mutant materials with the knockout of *GhCesA4*, *GhCesA7*, or *GhCesA8*, significant phenotypes such as dwarfing, and reduction in cotton bolls and fibers were also observed (Wen et al., 2022). Cuticular wax deposition is regulated in response to environmental conditions (Kosma et al., 2009; Go et al., 2014; Lee et al., 2016). *KCS*, as a key member of the fatty acid elongation complex enzyme system (FAE), plays a crucial role in the synthesis and accumulation of wax (Huang et al., 2023). Lu et al. (2020) which showed that silencing *GhFAR3.1* significantly decreased wax accumulation in leaves. In this study, the WD treatment significantly up-regulated the transcription levels of *GhKAS* and *GhFAR* genes in cotton (**Figure 10**). This result further confirms the phenomenon that the wax content in the fiber epidermis increases under water stress conditions. In conclusion, WD treatment inhibits the expression of genes related to sucrose and cellulose synthesis, reduces the content of sucrose and cellulose in fibers, and simultaneously induces the expression of wax synthesis genes, leading to excessive accumulation of wax. Ultimately, this results in a decline in the quality of cotton fibers. These findings provide an important basis for a deeper understanding of the physiological and molecular mechanisms of cotton fiber development under water stress. They also identify key regulatory targets and research directions for the cultivation of new cotton varieties that possess both drought resistance and excellent fiber quality.

## 5 Conclusions

WD treatment significantly reduces the sucrose content in cotton fibers, and this change profoundly affects the development process of cotton fibers. As a key substrate for cellulose synthesis, the decrease in the content of sucrose leads to a shortage of raw materials for cellulose synthesis. The thickness of the cell wall under WD treatment decreases by about 10%, and this structural change directly weakens the physical strength of cotton fibers. meanwhile, water stress triggers the defense response mechanism of cotton plants, leading to the formation of a thicker cuticular wax layer on the surface of the fibers. Although the excessive deposition of these substances on the fiber surface enhances the stress resistance of the fibers to a certain extent, these morphological changes may cause a decrease in fiber strength and elongation, thus having a negative impact on fiber quality. These findings provide valuable insights into the mechanisms driving fiber quality decline under drought conditions. Therefore, when plant breeders select drought-tolerant varieties, they cannot merely focus on the drought resistance of cotton, but also need to comprehensively consider the sucrose and wax properties of the fibers. In breeding practice, molecular marker-assisted selection technology can be used to screen cotton germplasm resources that can maintain a high sucrose metabolism level and reasonable wax synthesis regulation under WD conditions. Thus, it is expected to cultivate new cotton varieties that are both drought-tolerant and can maintain excellent fiber quality.

## Data Availability

The original contributions presented in the study are included in the article/supplementary material. Further inquiries can be directed to the corresponding authors.
