# Supplementary material for: Water stress reduces cellulose deposition in the cell wall and increases wax content, resulting in decreased fiber quality
Source: Front Plant Sci. 2025 Jul 1;16:1611390. doi: 10.3389/fpls.2025.1611390 (PMC12261924; doi:10.3389/fpls.2025.1611390)
Supplement: Supplementary file 1 [file SupplementaryFile1.docx]

**Supporting Information**

**Figure S1**

**The wax content of the fiber epidermis.**





SD127, Shidamian 217. XC7, Xincaimian 7.

**Figure S2** Determination of growth parameter indicators (2023-2024)





SD217, Shidamian 217. XC7, Xincaimian 7. WW, well-watered; WD, water-deficit.

**Table S1**

Primers for poly-­ubiquitin and target genes in quantitative real-time PCR program.

| Gene name | Gene ID | Primer sequence (5′−3′) | Tm (℃) |
| --- | --- | --- | --- |
| *GhUBQ7* |  | Forward: GAAGGCATTCCACCTGACCAAC  Reverse: CTTGACCTTCTTCTTCTTGTGCTTG | 58.5  59.2 |
| *GhSuSyA* | *Gh_D05G0363* | Forward: CCCACGGATACTTCGCTCAAGAC  Reverse: GAGGGGTGATGTTGAGTCCTTGTT | 61.3  59.6 |
| *GhSuSyB* | *Gh_D06G0832* | Forward: CCGTGCCTTGGAGAACGAGAT  Reverse: GCGTCAGGGAGAAGTCTGGTAAT | 59.5  59.5 |
| *GhSuSyC* | *Gh_D06G0825* | Forward: GTGAGCGTTTGGGTGAATCT  Reverse: GTGCTTGATTTCCGGCCTCT | 55.4  57.5 |
| *GhSuSyD* | *Gh_D13G2037* | Forward: ACGACCACAACAAGCCAATTCTAT  Reverse: CTCCGATCTCCACCGACAACTA | 59.2  59.5 |
| *GhCesA4* | *Gh_A08G0421* | Forward: CAGGAGATGAAGAAGATG  Reverse: TTCCATTCTTCGCTTCCG | 53.4  52.6 |
| *GhCesA7* | *Gh_A05G3965* | Forward: GAGGATGTGGATGATATCG  Reverse: CCATAAGCAAGAGAGCTTC | 53.1  53.1 |
| *GhCesA8* | *Gh_A10G0327* | Forward: TAAGGTTGAAAGAGAGGCTG  Reverse: TTCACATATGACTGAAGTG | 53.4  53.0 |
| *GhKAS* | *Gh_A05G1257* | Forward: CCAACAAAAAGCAGCAGCACGA  Reverse: GGCTGAAAATGCCACAACCGAG | 59.3  59.3 |
| *GhLACS* | *Gh_A07G1181* | Forward: CCCACAGTTTGGTTTGTGTGCC  Reverse: ATTGCTTTCAGCCGTTGAGCC | 60.0  59.5 |
| *GhFAR* | *Gh_D07G1069* | Forward: GGGGTTGTTGATGTCTTGCC  Reverse: GAAGGCATTCCACCTGACCAAC | 59.5  59.8 |
| *GhWSD* | *Gh_A07G1263* | Forward: TGGGGATAACACAGGCTGGTCT  Reverse: AGCAAAGTTGATCGGAGGCGAA | 60.7  59.6 |
